# Ocular Lesions in the Inmates of Leprosy Rehabilitation Centre

**Published:** 2006-09

**Authors:** S. C. Reddy, B. D. Raju

**Affiliations:** 1 *Department of Ophthalmology, Rangaraya Medical College, Kakainada, Andhra, Pradesh, India;*; 2 *Department of Ophthalmology, International Medical University Clinical School, Seremban, Negeri sembilsan, Malaysia;*; 3 *Zonal Leprosy Officer, National Leprosy Eradication Program, Kakinada, Andhra, Pradesh, India*

**Keywords:** ocular lesions, leprosy, lagophthalmos, corneal anaesthesia, iridocyclitis, cataract

## Abstract

A detailed eye examination of 145 inmates of a leprosy rehabilitation centre was done to determine the prevalence of ocular involvement. Age, gender of patients, type and duration of leprosy, systemic disabilities were noted. The mean age of patients was 45.8 years (range 19-70 years); 72.4% were males; 55.2% were suffering from paucibacillary leprosy. The mean duration of leprosy was 18.2 years in multibacillary type and 13.1 years in paucibacillary type. Ocular lesions related to leprosy were seen in 85.5% of patients; more often in multibacillary leprosy (92.3%). Corneal changes (80.7%) were the most frequently observed lesions followed by eye lid lesions (48.2%). Potentially sight threatening lesions such as lagophthalmos (23.4%), cornealanaesthesia (43.4%), and iridocyclitis (8.9%) were seen in both types of leprosy. Nine out of 26 (34.6%) patients with history of erythema nodosum leprosum reaction showed eye changes related to this reaction. Blindness in one eye due to lesions related to leprosy was seen in 2.7% of eyes. Age related cataract was the most common cause of blindness in patients of leprosy. The prevalence of ocular lesions was found to be high in the inmates of leprosy rehabilitation centre, and they were seen more frequently in patients with longer duration of the disease. Potentially sight threatening lesions were more often associated with systemic disabilities in these patients.

## INTRODUCTION

Leprosy, caused by *Mycobacterium leprae*, affects skin, nasal mucosa, peripheral nerves, anterior segment of the eye, and results in the disabilities and blindness if not treated in-time. Based on the clinical appearance of skin lesions, involvement of nerves and number of lepra bacilli in skin biopsy, the disease is classified as multibacillary (MB) or lepromatous type with a markedly impaired cellular immunity and very high bacillary load, paucibacillary (PB) or tuberculoid type with a relatively intact cellular immune function and low bacillary load, and borderline type which lies between two extremities with a variable degree of cellular immunity and may shift into either type during leprosy reactions. Leprosy remains a significant health problem in many countries worldwide, and India is one of them with high prevalence of registered patients (5 per 10,000 population) (1).

Leprosy affects the eye in four ways (2): 1) by direct invasion of lepra bacilli into eye structures (keratitis, iridocyclitis, scleritis, episcleritis); 2) secondary to involvement of facial nerve (lagophthalmos) and ophthalmic division of trigeminal nerve (corneal anaesthesia); 3) hypersensitivity reaction to the antigenic substances released in the breakdown of lepra bacilli (iridocyclitis, scleritis, episcleritis); 4) secondary to changes in the skin and support tissue of the lids, tear drainage system (madarosis, trichisis, entropion, chronic dacryocystitis).

Only few studies are available in the literature on the prevalence of ocular lesions in patients from rehabilitation centre (3), leprasorium (4), leprosy villages (5). The present study was conducted, with the collaboration of National Leprosy Eradication Program department, to determine the prevalence of ocular involvement in the inmates of a leprosy rehabilitation centre located in the outskirts of Kakinada town in Andhra Pradesh state, India, and to provide medical and surgical treatment for the needy patients.

## PATIENTS AND METHODS

All the inmates were examined in the rehabilitation centre itself. After explaining the purpose and conduct of the study, verbal consent was taken. Age and gender of patients, type and duration of the disease, systemic disabilities (absorption of fingers/toes, contracture of fingers, hand deformities, trophic ulcers on the feet, depression of nose) were noted. All patients with more than ten years diseases completed dapsone monothearpy, and the rest completed the multi drug treatment at the time of examination. A detailed eye examination was done by ophthalmologist. After taking the history of eye problems, visual acuity was tested on Snellen E chart at 6 meters distance in a well illuminated room. Those with vision less than 6/6 were tested again using pinhole or with spectacles in patients using glasses, to see for further improvement of vision. Detailed examination of the ocular adnexa (eyebrows, eyelids, lacrimal sac), anterior segment of the eye (conjunctiva, sclera, cornea, anterior chamber, iris, pupil, lens) was done with torch light and binocular loupe (Eagles Vision 1.75 X).

Lagophthalmos was tested by asking the patient to close the eyelids gently and any exposure of sclera/cornea was noted. The presence or absence of Bell’s phenomena was noted for consideration of treatment in these patients. Corneal sensation was tested with a clean fine cotton whip. If there was lagophthalmos preventing blink reflex, they were asked about subjective sensation of touch on the cornea. Intraocular pressure was measured with Schiotz tonometer under topical anaesthesia (xylocaine eye drops 4%). Then, both pupils were dilated with tropicamide eye drops (1%) and fundus examination was done with direct ophthalmoscope in a semi dark room. All the findings were documented on a proforma for analysis.

Patients requiring refraction and slit lamp examination were further evaluated in the eye clinic of the teaching general hospital of the medical college. Patients requiring medical treatment were treated at the centre itself. Those requiring surgery for lagophthalmos and cataract were admitted in the general hospital and operated lateron. This study was carried out over a period of one year.

The following definitions were used in this study: 1) corneal sensation normal - when there is spontaneous blinking/patient feels the sensation of touch; corneal sensation diminished (hypoesthesia) - when there is delayed blinking/patient feels less sensation of touch; corneal sensation absent (anaesthesia) - when there is no blinking/patient does not feel sensation of touch; 2) chronic iridocyclitis - history of redness, pain and diminution of vision in the eye, small irregular pupil with posterior synechiae/iris atrophy; 3) complicated cataract - evidence of past iridocyclitis with lenticular opacity reducing the vision to less than 6/18; 4) refractive error - visual acuity less than 6/6 which improves with pinhole/ glasses; 5) WHO categories of visual impairment (6): no visual impairment (6/6-6/18), visual impairment (<6/18-6/60), severe visual impairment (<6/60-3/60), blind (<3/60-perception of light).

Lagophthalmos, exposure keratitis, corneal anaesthesia, corneal ulcer/opacity in the pupillary area, chronic iridocyclitis, were considered as potentially sight threatening (PST) lesions. Others lesions such as nodules/infiltration of eyebrows/eyelids, superficial keratitis, corneal ulcer/opacity in the periphery were considered as academic lesions since they usually do not cause loss of vision.

## RESULTS

One hundred and forty five patients were examined, of whom 72.4% were males; the mean age of patients was 45.8 years (range 19-70 years); 55.2% were suffering from PB leprosy. The mean duration of the disease in MB patients was 18.2 years (range 6-36 years) and in PB patients 13.1 years (range 5-30 years). There was no patient with border line leprosy. Ocular lesions, at least one pathology in the eye, related to leprosy were seen in 124 (85.5%) patients; 98 of theses patients (79%) were suffering from the systemic disease for more than 10 years. Potentially sight threatening lesions were seen in 72 (49.6%) patients; some of these patients had more than one eye lesion.

Visual acuity of 290 eyes (145 patients) at presentation is shown in Table 1. The eyes are taken into consideration because vision may be good in one eye, and poor in the other eye of the same patient. Blindness due to lesions related to leprosy was seen in 8 eyes (2.7%). In the remaining 22 eyes, age related cataract was responsible for vision less than 3/60. Corneal lesions were more frequently seen in both types of leprosy, followed by eyelid lesions (Table 2). One or more eye lesions were observed in one or both eyes of these patients. Hence, the total number of lesions shown in the table are much more than the number of patients examined. Lateral tarsorrhaphy was performed in all the patients with exposure keratitis and prophylactic topical antibiotics and lubricants were given to prevent corneal ulceration. Lagophthalmos patients with good Bell’s phenomenon were advised lid excercises in addition to topical lubricants . Ectropion of lower lid was corrected by lateral tarsal strip procedure. Potentially sight threatening lesions were more often associated with systemic disabilities - lagophthalmos in 29 of 34 (85.3%) patients, iridocyclitis in 11 of 13 (84.6%) patients.

**Table 1:** Visual acuity at presentation in 290 eyes (145 leprosy patients)

WHO categories	Level of vision	MB leprosy (n=160)	PB leprosy (n=130)	Total (%) (n=290)

No visual impairment	6/6 – 6/18	77	75	152 (52.4%)
Visual impairment	6/18 – 6/60	30	58	88 (30.3%)
Severe visual impairment	6/60 – 3/60	8	12	20 (6.9%)
Blind	3/60 – PL	15	15	30 (10.3%)

PL, perception of light.

**Table 2:** Ocular lesions related to leprosy

Ocular lesions	Multibacillary leprosy (n=65)	Paucibacillary Leprosy (n=80)	Total (n=145)	Percentage

Eyebrows	46	2	48	33.1%
Total madarosis	22	1	23	15.9%
Partial madarosis	16	1	17	11.7%
Infiltration	5	-	5	3.4%
Nodules	3	-	3	2.0%
Eyelids	44	26	70	48.2%
Total madarosis	15	1	16	11.0%
Partial madarosis	5	2	7	4.8%
Infiltration	3	-	3	2.0%
Nodules	2	-	2	1.4%
Lagophthalmos	16	18	34	23.4%
Unilateral	9	9	18	12.4%
Bilateral	7	9	16	11.0%
Ectropion of lower lid	3	5	8	5.5%
Facial Palsy	1	1	2	1.4%
Chronic conjunctivitis	3	5	8	5.5%
Episcleritis	2	-	2	1.4%
Cornea	57	60	117	80.7%
Corneal anaesthesia	30	33	63	43.4%
Corneal hypoesthesia	15	20	35	24.1%
Exposure keratitis	5	3	8	5.5%
Central corneal ulcer	-	1	1	0.7%
Leproma of cornea	1	-	1	0.7%
Healed pannus (opacity)	5	2	7	4.8%
Superficial keratitis	1	1	2	1.4%
Iris and Pupil	17	6	23	15.9%
Acute Iridocyclitis	1	-	1	0.7%
Chronic iridocyclitis	8	4	12	8.2%
Unilateral	6	3	9	6.2%
Bilateral	2	1	3	2.0%
Iris pearls	1	-	1	0.7%
Sluggishly reacting pupil	7	2	9	6.2%
Complicated cataract	4	1	5	3.4%

Among the ocular lesions which are not related to leprosy, cataract was the most common eye disease (43.4%) seen in our study (Table 3). Some of the patients had more than one ocular lesion. In patients who had unilateral aphakia, intracapsular cataract extraction was performed in the other eye and glasses were prescribed after six weeks post operatively. In other patients with mature cataract, standard extracapsular cataract extraction with posterior chamber intraocular lens implantation was performed. There were no significant post operative complications in these patients. Nine out of 26 (34.6%) patients with history of erythema nodosum leprosum (ENL) reaction showed one or more eye changes related to this reaction (Table 4).

**Table 3:** Ocular lesions which are not related to leprosy in 145 patients

Ocular lesions	Number	Percentage

Immature cataract	52	35.8%
Mature cataract	10	6.9%
Tr. Cat. with adherent leukoma	1	0.7%
Aphakia	5	3.4%
Refractive error/Presbyopia	38	26.2%
Pterygium	7	4.8%
Bitot spots	6	4.1%
Open angle glaucoma	2	1.4%
Retinitis pigmentosa	1	0.7%
Synchiasis scintillans	1	0.7%
Chalazion	1	0.7%

**Table 4:** Leprosy related eye changes seen in erythema nodosum leprosum reaction patients in 52 eyes (26 patients)

Ocular lesion	Number	Percentage

Acutr iridocyclitis	1	1.9%
Chronic iridocyclitis	3	5.7%
Episcleritis	2	3.8%
Lagophthalmos	6	11.5%
Infiltration of eyebrows	5	9.6%
Infiltration of eyelids	3	5.7%
Nodules on eyebrows	3	5.7%
Nodules on eyelids	2	3.8%

## DISCUSSIONS

The prevalence of ocular involvement in leprosy depends on 1) type of leprosy; 2) duration of the disease; 3) patients received treatment or not; 4) presence or absence of other systemic disabilities; 5) number of reactions of leprosy; 6) newly diagnosed patients/hospitalized for other problems; 7) geographical pattern of general incidence of the disease; 8) expertise of the person examining the eyes i.e. ophthalmologist or medical officer/field staff working in leprosy; 9) type of ocular lesions included i.e. only lesions related to leprosy or other ocular findings also like cataract, glaucoma, pterygium etc; 10) analysis of lesions per eye or per person; 11) visual acuity taken for definition of blindness i.e. less than 3/60 or less than 6/60.

The prevalence of ocular lesions, lagophthalmos, corneal anaesthesia and anterior uveitis seen in our study is much higher than the three studies available on similar group pf patients (Table 5). This could probably be due to longer duration of the disease and associated systemic disabilities in majority of our patients.

**Table 5:** Comparative prevalence of ocular lesions and potentially sight threatening lesions in leprosy patients

Author	No.of Patients	Ocular lesions	Lagophthalmos	Corneal anaesthesia	Anterior uveitis

Sanjiv *et al*.(3)	85	78.2%	5.9%	–	5.9%
Mvogo *et al*.(4)	218	77.5%	10.1%	13.5%	2.3%
Mypet *et al*.(5)	456	48.0%	12.6%	–	2.2%
Present Study	145	85.5%	23.4%	43.4%	8.9%

A wide variation of prevalence of ocular lesions (8.3% to 90%), lagophthalmos (0.5% to 47%), corneal anaesthesia (0.5% to 60%) and anterior uveitis (0.7% to 50%) in leprosy patients has been reported in the literature from different countries (7). The major causes of visual disability and blindness in leprosy are corneal disease secondary to lagophthalmos and corneal anaesthesia, anterior uveitis and cataract. About 0.5 to 1% of leprosy patients would be blind owing to the disease, and an additional of 1 to 2% owing to age related cataract (1).

Shorey *et al*. (8) have reported the occurrence of lagophthalmos in 4.1% and iridocyclitis in 10.1% of patients who had ENL reaction, while the same were observed in 11.5% and 7.6% of similar patients respectively in our study. Rohatgi *et al*. (9) observed that occurrence of PST lesions was significantly more in patients with systemic disabilities (89%) as compared to patients without them. A similar findings were noted in our study. The limb deformities are easily identified by field workers and therefore can be used as important indicator for identifying the patients with PST lesions, and all such patients should be referred to ophthalmologist for further evaluation.

Though completion of appropriate course of anti leprosy treatment changes the status of the individual patient from “under active treatment” to “cured” in the registers of many leprosy control programs, it does not prevent subsequent development of disabling complications, particularly those of the eye (10, 11). The higher prevalence of ocular lesions after completion of treatment in our patients supports this hypothesis.

Although ocular leprosy is basically an anterior segment disease, lesions of posterior segment behind the oraserrata, probably due to direct spread from ciliary body, have been described (12) - 1) white, waxy, highly refractile deposits in the periphery of retina; 2) descrete, circular, waxy, occasionally pedunculated nodules on the retina extending into the vitreous. No such retinal lesions were seen in our patients.

## CONCLUSIONS

The prevalence of ocular lesions was found to be high in the inmates of leprosy rehabilitation centre, and they were seen more frequently in patients with longer duration of the disease. Potentially sight threatening lesions were more often associated with systemic disabilities in these patients. Improving the primary eye care training of health workers responsible for leprosy control, frequent eye check up by ophthalmologist, in-time treatment of PST lesions, and unrestricted use of cataract surgical services in the hospitals will reduce the prevalence of visual impairment and blindness in these patients.
